# Gramicidin inhibits human gastric cancer cell proliferation, cell cycle and induced apoptosis

**DOI:** 10.1186/s40659-019-0264-1

**Published:** 2019-11-25

**Authors:** Tingting Chen, Yong Wang, Yang Yang, Kaikai Yu, Xiangliao Cao, Fang Su, Huanbai Xu, Yongde Peng, Yudong Hu, Feng Qian, Zishu Wang

**Affiliations:** 1 Department of Medical Oncology, First Affiliated Hospital of Bengbu Medical College, Anhui Province Key Laboratory of Translational Cancer Research (Bengbu Medical College), 287 Changhuai Road, Bengbu, 233004 Anhui Province People’s Republic of China; 2 Department of General Surgery, Zhoupu Hospital affiliated to Shanghai Health Medical College, Shanghai, 201318 People’s Republic of China; 3 Engineering Research Center of Cell & Therapeutic Antibody, Ministry of Education, School of Pharmacy, Shanghai Jiao Tong University, 800 Dongchuan Road, Shanghai, 200240 People’s Republic of China; 4 Department of Endocrinology and Metabolism, Shanghai General Hospital, School of Medicine, Shanghai Jiao Tong University, Shanghai, 200080 People’s Republic of China

**Keywords:** Gramicidin, Proliferation, Apoptosis, Cell cycle arrest, SGC-7901

## Abstract

**Background:**

Gastric cancer is a common malignant tumor with high morbidity and mortality worldwide, which seriously affects human health. Gramicidin is a short peptide antibiotic which could be used for treating infection induced by bacteria or fungi. However, the anti-cancer effect of gramicidin on gastric cancer cells and its underlying mechanism remains largely unknown.

**Results:**

Gastric cancer cells SGC-7901, BGC-823 and normal gastric mucosal cells GES-1 were treated with different concentrations of gramicidin respectively. The results of CCK-8 experiment revealed cellular toxicity of gramicidin to cancer cells while cell colony formation assay showed that gramicidin significantly inhibited the proliferation of gastric cancer cells, but had little effect on normal gastric mucosal cells. In addition, the wound healing assay showed that gramicidin inhibited the migration of SGC-7901 cell. Meanwhile, apoptosis and cell cycle analysis revealed that gramicidin induced cell apoptosis with G2/M cell cycle inhibition. Furthermore, western blot analysis demonstrated that gramicidin down-regulated the expression of cyclinD1 and Bcl-2 as well as the FoxO1 phosphorylation.

**Conclusions:**

The current study illustrated the anti-tumor activity of gramicidin on gastric cancer cells, providing a possibility for gramicidin to be applied in clinical practice for the treatment of gastric cancer.

## Background

Gastric cancer (GC) is an important health problem worldwide and is the third leading cause of cancer-associated mortality [1]. Although the incidence rate of GC is decreasing, particularly in Asian countries [2], it still causes a large proportion of GC-related death in 5 years with rapid tumor development and poor prognosis. Currently, the clinical treatment of GC is comprehensively based on surgical resection and chemotherapy [3]. Although advanced chemotherapy may improve the survival rate, they have strong side effects [4]. Thus, finding effective drugs which have significant anti-gastric carcinoma effect are of great need undoubtedly.

Forkhead box protein O transcription factor family (FoxO) consists of four members, including FoxO1, FoxO3a, FoxO4 and FoxO6 [5] and plays a pivotal role in diverse biological functions such as cell cycle progression, cell apoptosis, glucose metabolism, and cell differentiation [6]. FoxO1 is widely expressed in tissues and regulates cyclin D1 and p21 [7] while elevated levels of phosphorylation of FoxO1 is associated with tumorigenesis [8]. Thus, FoxO1 is expected to be a potential target for anti-cancer drugs.

Gramicidin, a short peptide antibiotic derived from bacteria called *Bacillus brevis*, is composed of 15 l- and d-amino acid residues [9]. It is one of the very first antibiotics to be used in clinical practice [10] and has the most significant antibacterial effect on gram-positive bacteria, fungi and protozoa [11, 12]. Gramicidin mainly changes the permeability of the plasma membrane, and destroys the structure of the membrane causing Na+ influx/K+ efflux and depolarization of transmembrane potential [13, 14]. Gramicidin is often used as a membrane-active drug to study the structure and function of the membranes. Previous studies on renal cell carcinoma (RCC) found that gramicidin as an inhibitor of hypoxia-inducible factor (HIF), suppressed the cell growth and angiogenesis of renal cell carcinoma (RCC) which expresses the Von Hippel-Lindau (VHL) [15]. However, the function of gramicidin on gastric cancer has not been reported.

In this study, we focused on evaluating the possible anti-tumor effects of gramicidin in GC while investigating the possible underlying mechanism. Our study found that the proliferation of gastric cancer cells was decreased significantly upon gramicidin treatment with induction of apoptosis and inhibition of migration and cell cycle. Further analysis revealed that FoxO1 signaling and Bcl-2, cyclin D1 might be modulated.

## Materials and methods

### Gramicidin liquid preparation

10 μg gramicidin dissolved in DMSO to prepare a solution of 100 mmol/ml and stored at − 20 °C for long-term. Then the solution was diluted to different concentrations with DMSO for experiment or stored at − 20 °C.

### Cell lines and culture condition

Human gastric cancer cell lines SGC-7901 and BGC-823 were reserved by our laboratory. Human normal gastric mucosal cell lines GES-1 were bought from the cell bank of the Chinese Academy of Sciences (Shanghai, China). All the cell lines were cultured in RPMI 1640 medium supplemented with 10% fetal bovine serum (FBS) and 1% penicillin/streptomycin. The cells were grown in a humidified 37 °C incubator with 5% CO_2_.

### Reagents

Fetal bovine serum (FBS) was purchased from Hangzhou Sijiqing Biological Engineering Materials Co., Ltd. (Hangzhou, China). RPMI-1640, penicillin/streptomycin and phosphate buffer solution (PBS) were obtained from Gibco (Grand Island, USA). Dimethyl sulfoxide (DMSO) was bought from Sigma-Aldrich (St. Louis, USA). Wright-Giemsa Stain Kit was obtained from Nan Jing Jian Cheng Technology Company (Nanjing, China). Annexin V-FITC apoptosis detection kit and Cell counting kit-8 was provided by Beyotime Institute of Biotechnology (Shanghai, China). Antibodies against p-FoxO1, total- FoxO1, cyclin D1, Bcl-2 and GAPDH were purchased from Cell Signaling Technology, Inc. (Danvers, USA). HRP-conjugated secondary antibodies against rabbit and mice were supplied from Jackson ImmunoResearch Inc. (West Grove, USA). Methanol and ethanol were bought from Sinopharm Chemical Reagent Co., Ltd. (Shanghai, China).

### Colony formation assay

To observe the effect of gramicidin on proliferation of gastric cancer cells SGC-7901 and BGC-823 and normal gastric mucosa cells GES-1 cells. Cells in logarithmic growth phase were seeded in 6-well plates (500 cells/well), then cells were treated with DMSO (as a control group) and different concentrations of gramicidin (10 nM, 20 nM, 30 nM, 40 nM and 50 nM) with RPMI 1640 containing 10% FBS and incubated for 7–10 days until they reached optimal clones of 200 cells in each clone. The medium was discarded and methanol was added to each well for 20 min. Cells were stained with Giemsa for another 20 min and washed with water. The number of colonies in each well was counted. The data obtained were analyzed by Prism software (GraphPad Software, Inc.).

### Cell counting kit-8 assay

SGC-7901 cells (5000 cells/well) were cultured in a 96-well plate overnight and treated with different concentrations of gramicidin (0.1 μM, 0.3 μM, 1 μM, 3 μM, 10 μM, 30 μM and 100 μM). DMSO was used as a control. After 24 h, 10 μl of CCK-8 solution were added to each well for 2 h then the 450 nm-absorbance was measured by an microplate reader.

### Cell migration assay

The wound healing assay was applied to assess cell migration. Well-grown SGC-7901 cells were seeded in 12 well plates (5 × 10^5^ cells/well) in RPMI 1640 supplemented with 5% FBS and incubated under 37 °C and 5% CO_2_ condition for 24 h. When the cell fusion reached 90%, the wound was scratched with a sterile 200 µl tip. Then cells were washed with PBS for three times, and the corresponding concentration of gramicidin (0 μM, 0.3 μM, 1 μM and 3 μM) were added into each well. Photographs were taken at 0 h and 48 h respectively and the changes in the healing of scratches of each well were recorded. Finally, Image J was employed to compute the area covered by the cells, and the degree of decline in the healing rate after administration was calculated as compared with the DMSO solvent control group.

### Cell apoptosis assay

SGC-7901 cells in logarithmic growth phase were treated with trypsin without EDTA, and were seeded in 6-well plates with 10^6^ per well, then were incubated at 37 °C and 5% CO_2_ condition overnight. Gramicidin (0 μM, 0.3 μM, 1 μM and 3 μM) were added into corresponding well for 24 or 48 h. The cells were harvested, rinsed with pre-cooling PBS twice, then stained with Annexin V-FITC and PI for 5 min according to the manufacturer’s instructions. Flow cytometry was used to analyze the stained cells within 1 h. The data was analyzed by the FlowJo7.6 software.

### Cell cycle assay

SGC-7901 cells (5 × 10^5^/well) were cultured in 6-well plates and incubated with different concentrations of gramicidin (0 μM, 0.3 μM, 1 μM and 3 μM) for 24 h or 48 h. The cells were harvested and rinsed with cold PBS before cells were resuspended in 70% cold ethanol at 4 °C overnight. After centrifugation to remove ethanol, the cells were washed with pre-cold PBS for twice, treated with 10 μg/ml RNAse at 37 °C for 30 min and stained with 50 μg/ml PI solution in the dark for 5 min. Finally, samples were analyzed by flow cytometry (LSRFortessa TM X-20; BD Biosciences, San Jose, NJ, USA).

### Western blot

SGC-7901 cells (1 × 10^6^/well) were collected after being treated with gramicidin or DMSO for 48 h. Cells were then washed twice with ice-cold PBS. Then, loading buffer was added to the wells to extract protein. Protein lysates were centrifuged slightly and heated at 99 °C for 10 min. The lysates were fractionated by electrophoresis in 12% or 15% SDS-PAGE. Then protein lysates were transferred to nitrocellulose membranes and blocked with 5% non-fat milk in water for 2 h in room temperature. The membranes were incubated with primary antibodies at 1:1000 dilutions overnight at 4 °C with continuous agitation. Then the membranes were incubated with secondary antibody for 2 h at room temperature, followed by being washed three times. The proteins were visualized on the X-ray film. The densitometry analysis of band intensity was detected by ImageJ. The experiment was repeated three times independently.

### Data analysis and statistics

All values were presented as the mean ± SEM. Statistical significance of the results was performed with *t* test and one-way analysis of variance (ANOVA) using Graphpad Prism 5.0. Statistically significant P-values were defined as *P < 0.05 and **P < 0.01, ***P < 0.005. The chemical structure of gramicidin was presented by ChemDraw Professional 16.0 software.

## Results

### Cytotoxic effect of gramicidin on the gastric cancer

**Fig. 1:**
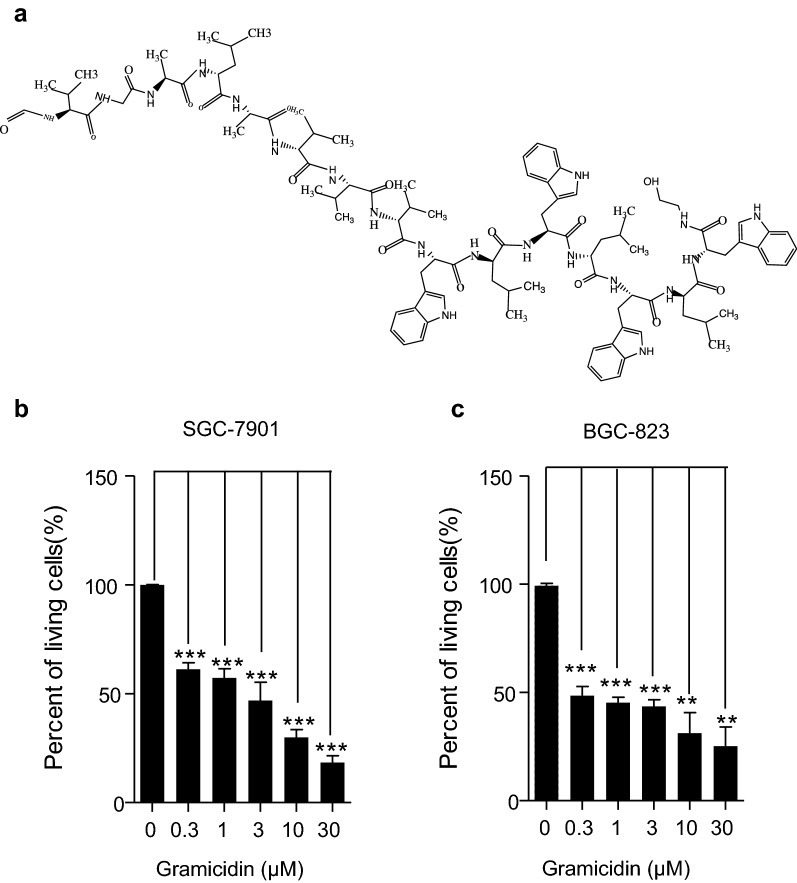
The chemical structure of gramicidin and its toxic effect on gastric cancer cells SGC-7901 and BGC-823 cells proliferation. **a** Chemical structure of gramicidin. The cell survival rate of **b** SGC7901 and **c** BGC-823 cells which were treated with 0, 0.3, 1, 3, 10 and 30 μM of gramicidin respectively in 96-well plate were quantitatively analyzed by CCK-8 assay. The results are shown as the mean ± SEM of three independent experiments (n = 3, *P < 0.05, **P < 0.01 and ***P < 0.001 vs. Control)

The chemical structure of gramicidin was shown in the Fig. 1a. To determine whether gramicidin exert cytotoxic effect on human gastric cancer SGC-7901 and BGC-823 cells, cell counting kit-8 assay was applied and the cells were treated with different concentrations of gramicidin for 24 h. As shown in Fig. 1b, c, the percent of living cells decreased significantly upon gramicidin treatment and gramicidin inhibited the proliferation of two different kinds of gastric cancer cells in a dose-dependent manner. The 50% inhibitory concentration (IC_50_) values of gramicidin, were 0.183 and 0.191 μM for the SGC-7901 and BGC-823 cells, respectively. In addition, results showed that SGC-7901 cells was more sensitive to the treatment of gramicidin.

### Effect of gramicidin on the cell proliferation

**Fig. 2:**
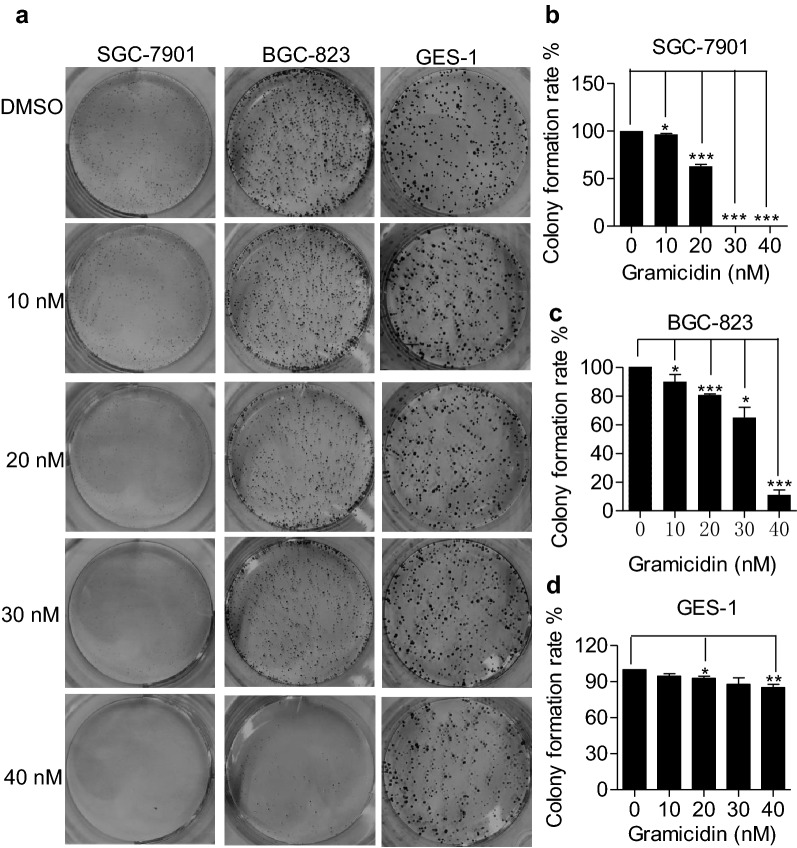
Inhibitory effect of gramicidin on gastric tumor SGC-7901, BGC-823 and GES-1 cells proliferation. Representative images of colonies in **a** SGC-7901, BGC-823 and GES-1 cells and quantification of the colony formation rate in **b** SGC-7901, **c** BGC-823 and **d** GES-1 cells from a six-well plate using colony formation assay while cells were treated with 0, 10, 20, 30 and 40 nM of gramicidin for 10 days, respectively. The results are shown as the mean ± SEM of three independent experiments (n = 3, *P < 0.05, **P < 0.01 and ***P < 0.001 vs. Control)

Cell proliferation plays important role in cancer development. We then investigated the anti-proliferative effect of gramicidin on human gastric cancer cells and colony formation assay was used. As shown in the Fig. 2a, cells were treated with gramicidin at various concentration for 10 days and the colony formation rate of SGC-7901 and BGC-823 cells decreased significantly. Quantitative analysis of the clone formation rate showed that gramicidin suppressed proliferative capacity of SGC-7901 and BGC-823 cells in a concentration-dependent manner (Fig. 2b, c). However, the proliferation of human gastric mucosal epithelial cells GES-1 was not affected by gramicidin when compared to the control group (Fig. 2d). Only when the concentration of gramicidin reached to 40 nM, the proliferation of the GES-1 cells was inhibited (P < 0.05). The above results suggested that the gramicidin could inhibit the proliferation of the gastric cancer cells SGC-7901 and BGC-823. As SGC-7901 showed a more sensitive pattern upon gramicidin treatment, we next evaluate further anti-tumor effect of gramicidin on GC using the SGC-7901 cells.

### Gramicidin induced the apoptosis of human gastric cancer cells

**Fig. 3:**
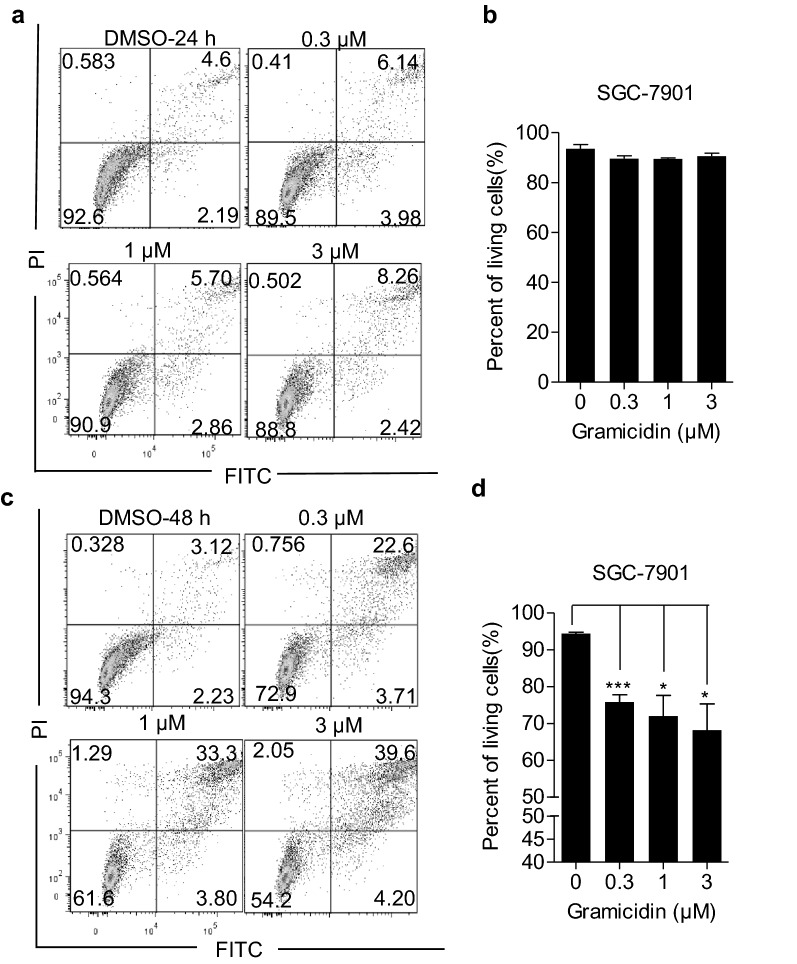
Gramicidin induced apoptosis in SGC-7901 cells. SGC-7901 cells were treated with 0, 0.3, 1 and 3 μM of gramicidin respectively for **a** 24 h and **c** 48 h, and were then subjected to Annexin V-FITC/PI staining, followed by flow cytometer analysis. Quantification of the percentage of apoptotic cells which were treated with gramicidin at various doses after treatment for **b** 24 h and **d** 48 h. The results are shown as the mean ± SEM of three independent experiments (n = 3, *P < 0.05 and ***P < 0.001 vs. Control)

Furthermore, to determine whether gramicidin induced apoptosis of human gastric cancer cells, Annexin V-FITC/propidium iodide (PI) double staining was performed. SGC-7901 cells were cultured with various concentrations of the gramicidin for 24 h or 48 h and flow cytometry was used to analyze the cell apoptosis. As demonstrated in Fig. 3a, c, gramicidin promoted apoptosis of SGC-7901 cells. The apoptotic rate of the gramicidin-treated cancer cells was increased remarkably in 48 h (Fig. 3b, d). When the cells were cultured with gramicidin for 48 h, the percent of living cells went down to 54.2% with 3 μM gramicidin treatment compared with the control group. These data indicated that gramicidin promoted the cell apoptosis of SGC-7901cells in a time and dose dependent manner.

### Gramicidin induced G2/M cell cycle arrest in human gastric cancer cells

**Fig. 4:**
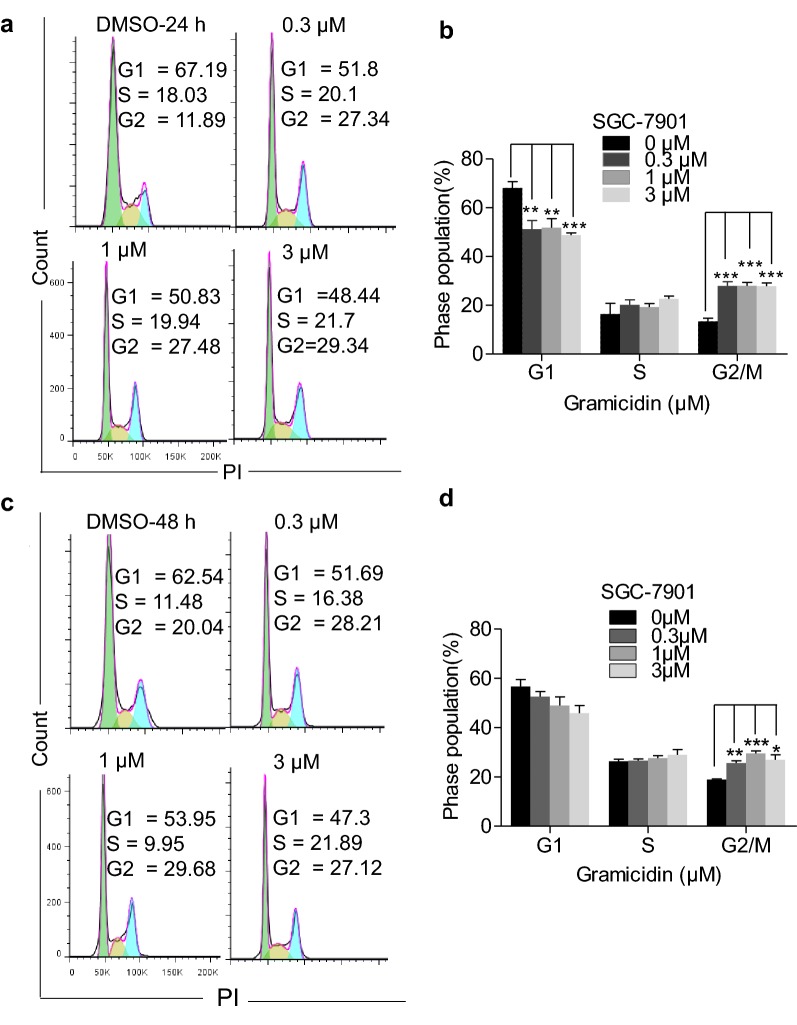
Gramicidin induced G2/M cycle arrest in SGC-7901 cells. Representative images of cell cycle distribution in **a** 24 h and **c** 48 h were shown. SGC-7901 cells were treated with 0, 0.3, 1 and 3 μM of gramicidin respectively. Quantification of the percentage of phase population (G1, S, G2/M) was showed in **b** and **d**. The results are shown as the mean ± SEM of three independent experiments (n = 3, *P < 0.05, **P < 0.01 and ***P < 0.001 vs. Control)

We next explored the effect of gramicidin on the cell cycle of human gastric cancer cells. Propidium iodide (PI) staining was used to detect the cell cycle distribution, followed by flow cytometry. As shown in Fig. 4a, c, the percentage of SGC-7901 cells with gramicidin treatment in the G1 phase was lower, compared to the percentage of control group in the G1 phase in 24 h and 48 h. Whereas statistical analysis revealed that the population of cells in the G2/M phase was significantly increased in SGC-7901 cells in 24 h and 48 h (Fig. 4b, d). These results demonstrated that gramicidin could arrest cell cycle at G2/M phase.

### Gramicidin inhibited the migration of SGC-7901 cell

**Fig. 5:**
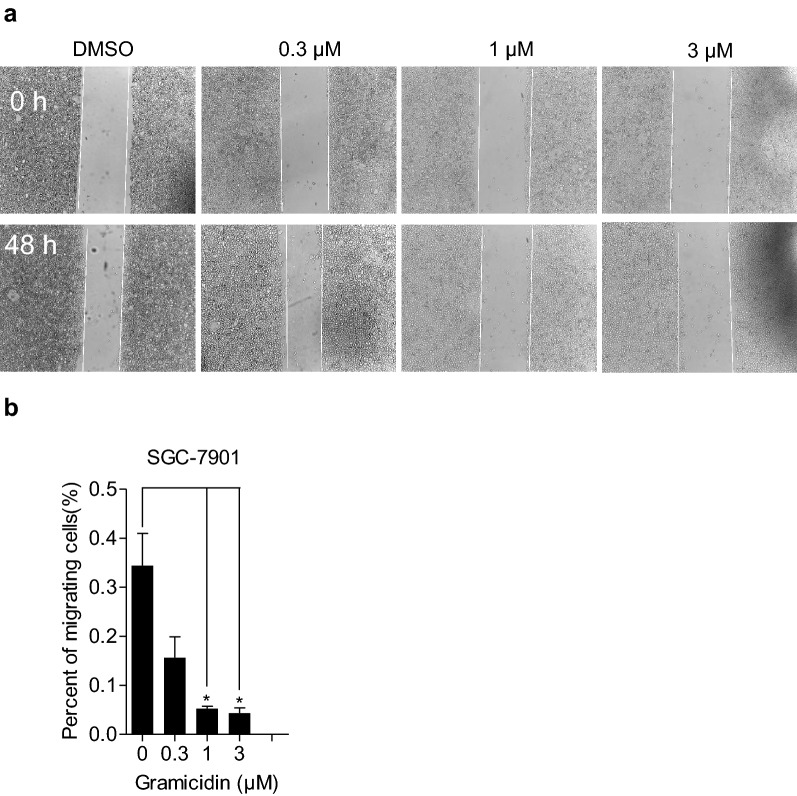
Gramicidin inhibited the migration in SGC-7901 cells. **a** Typical images of wound healing analysis in SGC-7901 cells treated with indicated dosage of gramicidin were compared at 0 h and 48 h. **b** The cell mobility of SGC-7901 cells which were treated with 0, 0.3, 1 and 3 μM of gramicidin respectively for 48 h in 12-well plate was quantitatively analyzed by image J software. The results are shown as the mean ± SEM of three independent experiments (n = 3, *P < 0.05 vs. Control)

To detect the effect of gramicidin on migration ability of SGC-7901 cells, we next performed the wound healing assay. The data showed that the migration area of SGC-7901 with gramicidin treatment were obviously lower than the area of the control groups (Fig. 5a). Moreover, quantification analysis revealed that gramicidin resulted in a dose-dependent decrease of inhibiting the cell migration (Fig. 5b). Thus, the results suggested that gramicidin inhibited the SGC-7901 cell migration.

### Gramicidin treatment downregulated the expression levels of Bcl-2, cyclin D1 and p-FoxO1

**Fig. 6:**
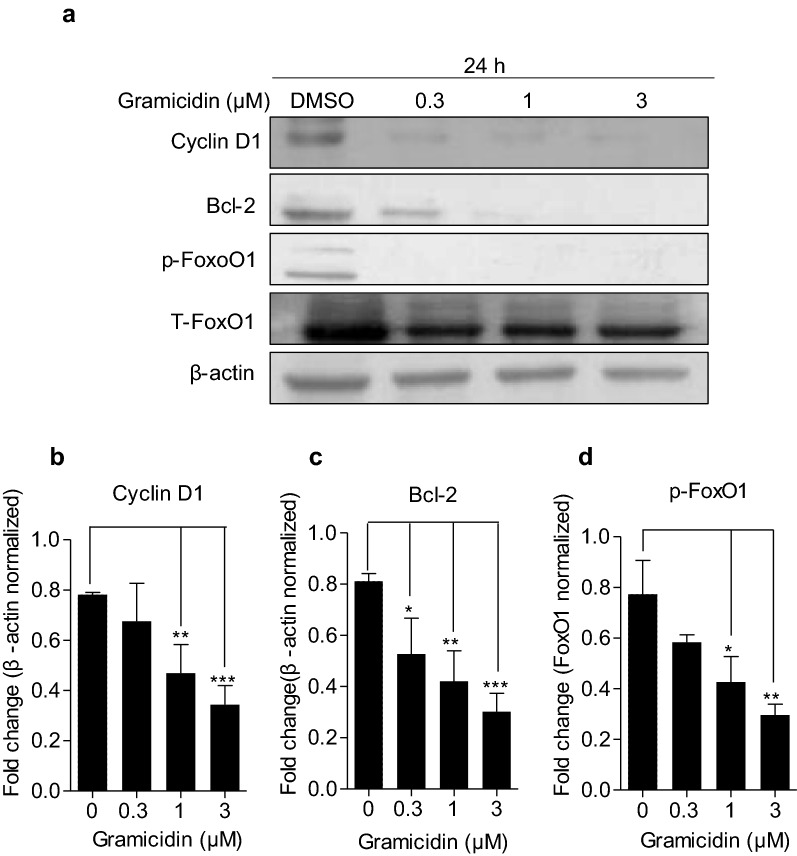
Gramicidin induced gastric cancer cell apoptosis by decreasing the expression of cyclin D1, Bcl-2 and p-FoxO1. **a** The expression of cyclin D1, Bcl-2 and p-FoxO1 in SGC-7901 cells which were treated with 0, 0.3, 1 and 3 μM of gramicidin respectively for 24 h were analyzed by western blot. β-actin was used as a loading control. The fold change of **b** Bcl-2 and **c** cyclin D1 to β-actin and **d** p-FoxO1 to total-FoxO1 were shown. The results are shown as the mean ± SEM of three independent experiments (n = 3, *P < 0.05, **P < 0.01 and ***P < 0.001 vs. Control)

Next, we investigated the possible mechanism underlying the anti-cancer effect of gramicidin. The expression of apoptosis-related proteins was detected by western blot analysis. Figure 6a revealed a remarkable decrease in the expression of cyclin D1 and anti-apoptotic protein Bcl-2 compared with control group. In addition, the expression of p-FoxO1 was significantly downregulated following different concentrations of gramicidin. The fold change of Bcl-2 and cyclin D1 to β-actin and p-FoxO1 to total-FoxO1 were shown respectively in Fig. 6b–d, revealing that the regulatory effect of gramicidin on the expression of apoptosis-related proteins was in a dose-dependent manner.

## Discussion

The current study evaluated the cell cytotoxicity of gramicidin and its anti-proliferation activity in human gastric cancer cells. Meanwhile, the migratory capacity and G2/M cell cycle were inhibited upon gramicidin treatment with significant cell apoptosis. Furthermore, our results showed that this influence could be possibly attributed to the decreased expression of cyclin D1 and Bcl-2 with down-regulated FoxO1 phosphorylation. Despite of the recent findings in GC treatment which brought new targets and possible therapies like PD-1/PD-L1 checkpoint inhibitors [16], there is still no specific and effective drug that can cure gastric cancer. Thus, it is an urgent problem to find low toxicity and effective drugs to induce cell apoptosis while inhibiting cell proliferation and migration of tumor cells. Adenocarcinoma in gastric cancer has a high frequency of occurrence. Both SGC-7901 and BGC-823 were adenocarcinoma and these two types of gastric cancer cell lines were chosen as tools for biological studies. SGC-7901 was obtained from moderately differentiated lymph nodes of metastatic adenocarcinoma, while BGC-823 was obtained from undifferentiated adenocarcinoma. Their different sensitivities to gramicidin suggested that the level of differentiation may affect the anti-cancer gramicidin and SGC-7901 from metastatic adenocarcinoma lymph nodes with moderate differentiation was more sensitive to gramicidin. Although the effect of cell proliferation could not be excluded, we used low concentration of serum for migration assay. Thus, our study illustrated the effect of gramicidin on inhibiting cell proliferation, migration and apoptosis induction of gastric cancer cells while suggesting that gramicidin could be served as potential candidate for GC treatment.

Apoptosis is the typical kind of programmed cell death, which refers to the self-regulated and orderly death of cells controlled by multiple genes in order to maintain the stability of internal environment [17]. Resistance to cell death often leads to tumorigenesis while targeting apoptosis has been regarded as one of the common method in controlling cancer progression [18]. Among complex pathways regulating cell apoptotic fate such as ER, mitochondrial and TNF/TNF-R mediated cell death, mitochondria driven cell death plays a critical role [19, 20]. The process of mitochondria driven apoptosis involves the activation, expression and regulation of a series of genes and proteins like transcription factors and apoptosis-related Bcl-2 family. Bcl-2 proteins family, including anti-apoptotic Bcl-2 and pro-apoptotic BAX, regulates apoptosis by controlling mitochondrial permeability with subsequent release of caspases [20–22]. Correspondingly, our data also showed that Bcl-2 decreased at various doses of gramicidin which is consistent with the mitochondrial apoptosis pathway, suggesting that gramicidin might exert its pro-apoptotic effect through targeting mitochondria. Of note, the cell counting kit-8 assay showed significant toxicity of gramicidin on cell survival at 24 h while the expression of Annexin V did not altered significantly, suggesting that gramicidin might not only affect apoptosis but other form of cell death like autophagy.

Beside the cell apoptosis, targeting cell cycle has advanced the chemotherapy for decades. Dysregulated cell cycle would cause uncontrolled cell proliferation while permitting cells obtain the ability to replicate and grow without limitation [23, 24]. Thus, targeting cell cycle with inhibited cell replication could arrest neoplastic process [25]. In cell cycle movement, cyclins play critical roles in triggering and promoting cell cycle from G1 to S and G2/M phases. It was reported that cyclin D1 could be involved in diverse cycle status. Typically, the function of cyclin D1, or G1/S-specific cyclin-D1, is to promote cell proliferation by combining and activating the G1 period characteristic of the cycle dependence protein kinase CDK4 [26]. G1 phase cycle suppressor protein (Rb) is phosphorylated, and the phosphorylated Rb protein is dissociated from the E2F transcription factor to which it binds. E2F transcription factors initiate transcription of genes that are involved in the cell cycle, thus promoting the cell cycle from the G1 phase to the S phase [27]. However, in our study, little inhibition in G1/S were observed while higher inhibition in G2/M cell cycle were detected, suggesting that gramicidin might affect a different signaling to regulate the expression of cyclin D1 since cyclin D1 was also involved in signal transcription like Wnt/GSK-3 beta pathway in which cyclin D1 functions as a downstream factor to regulate cell survival [28]. Similarly, sweroside and lanatoside C could arrest leukemia cells or gastric cancer cells at G2/M while lowering the expression of cyclin D1 [29]. Our study further confirmed that gramicidin regulated cyclin D1 expression with G2/M inhibition and apoptosis, implying that modulating cyclin D1 might serve as a potential anti-tumor target.

FoxO1 is a transcriptional factor that plays a key role in the development and progression of gastric cancer. More studies have confirmed that FoxO1 is involved in a variety of cellular pathways, such as proliferation, stress resistance, differentiation and apoptosis. Many research focused on its regulatory effect on cell cycle. For example, polo-like kinase 1 (PLK1) interacts and phosphorylates FoxO1, abrogating its inhibitory effects on cell survival, thus promoting G2/M cycle progression [30]. In proliferating cells, FoxO1 could be activated by cyclin-dependent kinase 1 (Cdk1) to affect the expression of PLK [31]. In light of these findings, numerous research has proposed that regulating FoxO1 could be applied in cancer therapy. Knock down of FoxO1 inhibited tamoxifen sensitivity in breast cancer cells [32]. Our study revealed the effect of gramicidin on FoxO1 phosphorylation. After gramicidin treatment, the phosphorylation of FoxO1 decreased with the increase of cell apoptosis, indicating that increasing FoxO1 activity might contribute to cellular apoptotic fate.

Previous studies have shown that systemic intravenous or intraperitoneal injection of gramicidin is fatal to mice. However, repeated injection of gramicidin into the tumor can block tumor growth without obvious toxicity [15]. Toxicity limits the clinical application of gramicidin and many other pre-clinical drugs [33]. Systemic administration is generally considered to be a necessary means of treatment for invasive tumors. Local administration sometimes is an effective treatment for non-resectable or metastatic tumors and can be given by special means [34, 35]. With the development of science and technology, we believe that the toxicity of gramicidin can be solved via structure modification or combination usage with other clinical anti-cancer drugs, and this chemical can be applied as a novel drug or leading chemical for the treatment of GC in the future.

## Conclusion

In summary, the antitumor effect of gramicidin on gastric cancer cells was preliminarily explored, in which gramicidin may inhibit cell proliferation and induce G2/M cell cycle block by down-regulating the FoxO1 and cyclinD1 while down-regulating tumor suppressor genes Bcl-2 to induce apoptosis. Combined with previous studies, gramicidin may have potential value as a clinical treatment drug for gastric cancer. The toxicity of drugs is one of the key problems to be solved. Meanwhile, the possible mechanism of its modulation of cell apoptosis merits further investigation.

## Data Availability

All data analyzed during this study are included in this article.

## Abbreviations

FoxO: Forkhead box protein O transcription factor family
RCC: renal cell carcinoma
HIF: hypoxia-inducible factor
VHL: Von Hippel-Lindau
FBS: fetal bovine serum
PBS: phosphate buffer solution
PI: propidium iodide
DMSO: dimethyl sulfoxide
Cdk1: cyclin-dependent kinase 1
